# ASO Author Reflections: Safety and Feasibility of Robot-Assisted Minimally Invasive Esophagectomy (RAMIE) with Three-Field Lymphadenectomy and Neoadjuvant Chemoradiotherapy in Patients with Resectable Esophageal Cancer and Cervical Lymph Node Metastasis

**DOI:** 10.1245/s10434-023-13137-8

**Published:** 2023-02-03

**Authors:** T. J. Weijs, S. van der Horst, R. van Hillegersberg

**Affiliations:** Department of Surgery, University Medical Center Utrecht, Utrecht, The Netherlands

## Past

Esophageal cancer remains a devastating disease, of which the incidence is increasing.^1^ The cornerstone of treatment with curative intent is surgical resection. However, in case of preoperatively confirmed lymph node metastasis to the neck, patients are generally precluded surgery. The concurrent palliative treatment approach is associated with a limited 1 year survival of 32–39%. An alternative treatment approach would be neoadjuvant chemoradiotherapy followed by robot-assisted minimally invasive esophagectomy (RAMIE) with three-field lymphadenectomy, including the cervical lymph nodes. Whether this would be feasible and safe for the Western population was unclear up to now.

## Present

This study shows that robot-assisted minimally invasive esophagectomy (RAMIE) with three-field lymphadenectomy is feasible in patients with resectable esophageal cancer presenting with cervical lymph node metastasis in a Western population.^2^ Moreover, postoperative complications are comparable to those reported after curative treatment by high-volume centers and randomized trials. Overall 1 year survival was higher (85%) than reported for palliative treatment approaches, though this was not a primary endpoint for this study. An important finding was the high rate of interval metastasis in patients presenting with concomitant celiac trunk lymph node metastasis as well as poorly differentiated tumors. Additionally, patients rarely had cervical lymph node metastasis contralateral to the preoperatively detected cervical lymph node metastasis.

## Future

The results of this study will be used to design a nationwide multicenter study. In this prospective cohort trial, patients with celiac trunk metastasis or poorly differentiated tumors will first receive a potent systemic therapy [fluorouracil + leucovorin + oxaliplatin + docetaxel (FLOT) combined with trastuzumab for Her2-Neu-positive tumors] combined with immunotherapye^3^ followed by restaging before entering the NODE treatment. The other patients will receive upfront the NODE protocol as described here. A bilateral cervical lymph node has been found unnecessary and will be replaced by a unilateral dissection. This study will compare the survival of patients who receive this treatment protocol with palliatively treated patients in the population.
